# Draft Genome Sequence of Salmonella enterica subsp. *enterica* Serovar Livingstone 1236H, a Desiccation-Resistant Strain That Poses a Salmonellosis Hazard in Low-Moisture Foods

**DOI:** 10.1128/MRA.01197-20

**Published:** 2021-01-07

**Authors:** Ahmed G. Abdelhamid, Yumin Xu, Ahmed E. Yousef

**Affiliations:** a Department of Food Science and Technology, The Ohio State University, Columbus, Ohio, USA; b Department of Microbiology, The Ohio State University, Columbus, Ohio, USA; University of Maryland School of Medicine

## Abstract

Salmonella enterica serovar Livingstone 1236H was isolated originally from peanut butter and represents a health risk in low-moisture foods. The current work presents the strain’s genome sequencing results, which show a 4,824,729-bp genome sequence and 4,435 protein coding sequences, including some that are involved in adaptation to low-moisture environments.

## ANNOUNCEMENT

Some nontyphoidal Salmonella enterica serovars survive well in low-moisture (LM) foods (1) and contribute to salmonellosis outbreaks in products such as nuts, peanut butter, and tahini (2). Salmonella enterica serovar Livingstone 1236H (formerly Salmonella enterica serovar Eimsbuettel 1236H [3, 4]) was isolated originally from peanut butter (5). Although *Salmonella* Livingstone strains were commonly isolated from human, food, or animal sources (https://www.ncbi.nlm.nih.gov/pathogens/isolates/#Livingstone), 1236H was reported only in peanut butter. Here, we report the first draft genome sequence of *Salmonella* Livingstone 1236H and provide an insight into the strain’s biological characteristics that are associated with its survival in an LM environment.

*Salmonella* Livingstone 1236H was grown in tryptic soy broth (BD, Sparks, MD) at 37°C for 24 h. The genomic DNA (gDNA) was extracted from a 1-ml culture (10^9^ CFU/ml) using a gDNA extraction kit (QIAamp DNA minikit; Qiagen, Germantown, MD), according to the manufacturer’s instructions. The purity and concentration of the gDNA were checked using a spectrophotometer (NanoVue Plus; Biochrom USA, Holliston, MA). DNA libraries were prepared from the extracted gDNA using a library preparation kit (Nextera DNA Flex; Illumina, Madison, WI) and indexed with a library indexing kit (Nextera DNA CD indexes; Illumina), following the manufacturer’s protocol. The concentration and sizing of the DNA libraries were measured using a fluorimeter (Qubit; Invitrogen, Waltham, MA) and a bioanalyzer (model 2100; Agilent Technologies, CA), respectively. *Salmonella* Livingstone’s DNA libraries were sequenced using an Illumina MiSeq platform (Food Microbiology Laboratory, The Ohio State University, Columbus, OH), yielding paired-end raw reads (2 × 300 bp). The quality of the raw reads (1,120,586 reads; 616.7 million bases; quality score, 35; *N*_50_, 728,389 bp; coverage, 128×) was checked using FastQC v0.11.9 software (6), and the adaptors were trimmed using the Generate FASTQ analysis module (Illumina). The reads were used for *de novo* genome assembly using SPAdes v3.10.1 (7), resulting in 30 contigs. The generated contigs of strain 1236H were ordered using progressiveMauve v2.4.0 (8) against the reference genome, *Salmonella* Typhimurium strain LT2 (the average nucleotide identity was 98.5% as determined by FastANI v0.1.2 [9]). The assembled genome of *Salmonella* Livingstone 1236H was annotated using the NCBI Prokaryotic Genome Annotation Pipeline (10), which revealed a genome size of 4,824,729 bp, a GC content of 52.1%, 4,435 protein coding sequences, 77 tRNAs, 16 rRNAs, and 13 noncoding RNAs (ncRNAs). All bioinformatics software was used with default settings.

*Salmonella* Livingstone 1236H belongs to sequence type 638 as determined by MLST v2.0 (11). The strain was originally serotyped as *Salmonella* Eimsbuettel 1236H using the Kaufmann-White scheme (Tom Hammack, personal communication); however, the whole-genome-based serotyping tool SeqSero v1.2 (12) classified the strain as serotype 7:d:1,w (Livingstone). Being adaptable to LM environments (4), the annotated genome sequence of strain 1236H was found to contain gene sets associated with desiccation resistance, such as those involved in potassium ion transport (*kdpABCDE*), glycine betaine/proline transport (*proP* and *proVWXYZ*), and trehalose biosynthesis (*otsAB*) (1).

### Data availability.

The genome sequence of *Salmonella* Livingstone 1236H was deposited at GenBank under accession number JACVCX000000000.1. The linked BioProject, BioSample, and SRA accession numbers are PRJNA661276, SAMN16049523, and SRS7317145, respectively.
